# Sobrepeso y obesidad: factores familiares, dietéticos y de actividad física en escolares de una institución educativa de estrato medio-alto en Cali, Colombia

**DOI:** 10.7705/biomedica.6396

**Published:** 2022-05-01

**Authors:** Adela Herrera, Consuelo Sarmiento

**Affiliations:** 1 Grupo de Investigación de Cuidado en Salud, Programa de Enfermería, Universidad Libre, Cali, Colombia Universidad Libre Universidad Libre Cali Colombia; 2 Departamento de Pediatría, Facultad de Salud, Universidad del Valle, Cali, Colombia Universidad del Valle Universidad del Valle Cali Colombia; 3 Programa de Enfermería, Universidad Libre, Cali, Colombia Universidad Libre Universidad Libre Cali Colombia

**Keywords:** sobrepeso, obesidad pediátrica, ejercicio físico, clase social, estado nutricional, Overweight, pediatric obesity, exercise, social class, nutritional status

## Abstract

**Introducción.:**

Colombia es un país con problemas de sobrepeso y obesidad que, en muchas ocasiones, se deben a malos hábitos alimenticios.

**Objetivos.:**

Describir la prevalencia del sobrepeso y la obesidad, así como los factores familiares, dietéticos y de actividad física en un grupo de escolares entre los 6 y los 9 años de edad pertenecientes al estrato socioeconómico 4 en Santiago de Cali, Colombia.

**Materiales y métodos.:**

Se hizo un estudio descriptivo de corte transversal en 150 niños de una institución educativa. Los datos sociodemográficos se recolectaron con un instrumento validado previamente. Para el diagnóstico del estado nutricional, se tomaron medidas antropométricas y se analizaron con el programa Anthro Plus de la Organización Mundial de la Salud (OMS). Se detectaron 62 niños con sobrepeso u obesidad.

**Resultados.:**

En cuanto al diagnóstico nutricional, 42 (28 %) niños tenían sobrepeso y 20 (13 %), obesidad. El 78 % pertenecía a los estratos socioeconómicos 4 y 5. En cuanto a las características familiares, el 47 % tenía un solo hermano y el 25 % correspondía a hijos únicos; el 76 % de las madres y el 70 % de los padres eran profesionales; el 95 % de los niños no hacía actividad física importante.

**Conclusiones.:**

En el análisis de la asociación entre algunas variables, se evidenció la relación entre el estado nutricional y el número de hermanos; los escolares con mayor prevalencia de sobrepeso u obesidad, con mayor frecuencia eran hijos únicos o tenían solo un hermano. Se estableció asociación con la edad de las madres, especialmente entre los 41 y los 50 años, la escolaridad de los padres, el hecho de tener una ocupación o trabajo fuera del hogar, y la pertenencia a los estratos socioeconómicos 4 y 5.

## Abstract (en)

**Introduction:**

Colombia is a country with overweight and obesity problems, which, in many cases, are due to bad eating habits.

**Objective:**

To describe the prevalence of overweight and obesity, as well as family, dietary, and physical activity factors in a group of schoolchildren between 6 and 9 years old from socioeconomic level 4 in Cali, Colombia.

**Materials and methods:**

This was a descriptive cross-sectional study of 150 children from an educational institution in Cali. To collect the sociodemographic data we used a previously validated instrument; the anthropometric measurements were analyzed to diagnose the nutritional status in the World Health Organization (WHO) Anthro Plus program program. A total of 62 children were identified as overweight and obese.

**Results:**

Regarding the nutritional diagnosis, 42 (28%) children were overweight and 20 (13%) were obese; 78% belonged to socioeconomic levels 4 and 5. As for the family characteristics, 47% had only one sibling and 25% were only children; 76% of the mothers and 70% of the fathers were professionals; 95% of the children did not do significant physical activity.

**Conclusions:**

Our analysis showed an association between the nutritional status and the number of siblings; schoolchildren with a higher prevalence of overweight or obesity tended to be only children or have just one sibling. We also found associations between mothers’ age, especially at ages 41-50 years, parents’ schooling and whether they had an occupation or job outside the home, as well as belonging to socioeconomic levels 4 and 5.

Según la Organización Mundial de la Salud (OMS), la obesidad es el aumento anormal de la grasa corporal ^1^^,^^2^. El sobrepeso se define como un aumento global de peso ^3^, y la obesidad ha pasado a ser una alteración compleja en la que influyen factores físicos, psicológicos, biológicos, genéticos, sociales, económicos, culturales y ambientales.

A nivel mundial, el 10 % de los menores entre los 5 y los 17 años tiene sobrepeso y entre el 2 y el 3 % es obeso ^4^^,^^5^. En el 2008, casi el 17 % de los niños y adolescentes entre los 2 y los 19 años eran obesos y, en Colombia, la Encuesta Nacional de la Situación Nutricional (ENSIM, 2015) ^6^, registró 24,4% de exceso de peso en escolares, incluidos el sobrepeso (16,8 %) y la obesidad (7,6 %).

Las tasas de prevalencia varían considerablemente entre regiones, siendo mayores en América. La problemática es cada vez mayor y ha llevado a la OMS a considerar esta situación como una epidemia global ^7^. A nivel mundial, entre 40 y 50 millones de niños en etapa escolar son obesos y 200 millones están con sobrepeso, lo que representa el 10 % de todos los niños en el mundo ^8^.

Las causas de morbimortalidad en Colombia han cambiado con el aumento de las enfermedades crónicas no transmisibles y la modificación de los estilos de vida, la adopción de patrones inadecuados de alimentación, y la aparición de enfermedades como el sobrepeso y la obesidad infantil ^9^.

Según la OMS, el diagnóstico de sobrepeso en niños mayores de 5 años con base en la actualización de las curvas existentes, se establece cuando el índice de masa corporal (IMC) está por encima de una desviación estándar y menos de dos, y el de obesidad, cuando está por encima de dos desviaciones estándar. Este IMC elevado puede determinar la adiposidad en la vida adulta y se asocia con una elevada morbimortalidad en estas poblaciones ^10^.

Es importante y necesario tener datos actualizados y propios de la región para poder establecer estrategias de manejo que arrojen resultados y contribuyan a disminuir y prevenir este fenómeno que repercute en la aparición de enfermedades en la edad adulta ^11^. La alteración de los factores dietéticos, acompañada de un patrón disminuido de actividad física con mayor sedentarismo, se cuentan entre las causas más importantes del aumento de la obesidad ^4^^,^^9^^,^^12^.

Aunque hay muchos estudios sobre el aumento de este problema ^13^, es importante insistir en la intervención desde la infancia y trabajar sobre las causas de la situación, involucrando a los padres, las instituciones educativas, los docentes, la industria y los medios de comunicación, con el fin de articular las acciones ^12^ y contribuir a disminuir los gastos que este problema de salud pública genera a la sociedad y a las familias. Deben dejarse de lado las acciones aisladas y poco estructuradas que apenas brindan información sobre la promoción de estilos de vida saludables, desconociendo el contexto en los que se desarrollan los niños ^7^^,^^14^.

En el presente estudio, se describió la prevalencia de sobrepeso y obesidad en un grupo de escolares, así como los factores sociales, familiares, dietéticos y de actividad física, abordándose este problema de salud pública en un contexto real.

## Materiales y métodos

Se hizo un estudio descriptivo de corte transversal en una población compuesta por 150 niños entre los 6 y los 9 años de los grados segundo a quinto de primaria, escogidos por conveniencia. Para el cálculo de la muestra, se tuvo en cuenta la prevalencia de sobrepeso y obesidad en Colombia.

Se detectaron 42 niños con sobrepeso y 20 con obesidad, y se determinaron el sexo, el grado escolar, la edad de la madre, el número de hermanos, la ocupación y profesión de los padres, y el estrato socioeconómico. Para aceptar la hipótesis nula, se consideró un nivel de significación del 5 % con el fin de garantizar una mayor confiabilidad de los resultados.

### Análisis estadístico

La institución educativa se seleccionó entre dos colegios de estrato medio a alto de Cali, teniendo en cuenta su mayor prevalencia de sobrepeso y obesidad en niños con mayores índices de riqueza ^15^. Siguiendo los datos de la última encuesta en Colombia ^6^, el estrato socioeconómico se estableció con base en la zona en donde se encontraban ubicados el colegio y la vivienda de los niños.

Para determinar los factores sociales y familiares de los escolares con sobrepeso y obesidad, se preguntó a cada participante sobre las siguientes características sociales y familiares, las cuales se confirmaron en los archivos de la institución educativa: edad de los padres, profesión, ocupación, estrato socioeconómico de la vivienda y número de hermanos, tiempo frente a pantallas y actividad física diaria. Se preguntó, también, por el consumo de alimentos en la dieta diaria y su frecuencia.

La entrevista se hizo utilizando ejemplos y material didáctico apropiado para explicar de manera clara en el lenguaje propio de los niños todo lo relacionado con el consumo, la frecuencia y el tipo de alimentos; también, se utilizaron imágenes de diferentes tamaños para precisar el consumo, para lo cual se contó con la ayuda de una nutricionista experta, en tanto que una enfermera profesional tomó las medidas antropométricas de peso y talla; estas profesionales estaban debidamente entrenadas. En la toma de las medidas antropométricas, se emplearon balanzas digitales calibradas con una precisión de 0,1 kg y un tallímetro fijo muy bien colocado para disminuir los sesgos de información y lograr una mayor confiablidad de los datos. Esta información se diligenció en un cuestionario de frecuencia de consumo de alimentos validado en un estudio previo de una de las investigadoras ^16^.

Para comparar las proporciones en el cálculo de la frecuencia de los grupos de alimentos, se utilizó la prueba de ji al cuadrado; la frecuencia de alimentos se estableció como diaria, semanal o mensual y estos se clasificaron en 17 grupos tomados como referencia de las ENSIM 2015 ^6^ y 2010 ^17^.

Se hizo un análisis de correlación y asociaciones para identificar los factores sociales, familiares y dietéticos, y su relación con la obesidad y el sobrepeso, además de asociaciones estadísticas de algunas de las variables del estudio utilizando la prueba de independencia con un alfa de 0,05 para obtener resultados de mayor confiabilidad. Para el análisis de la información antropométrica, los datos de cada participante se ingresaron al programa AnthroPlus de la OMS ^18^.

En cuanto a la valoración del estado nutricional de los escolares, se utilizaron las guías del Ministerio de Protección Social de Colombia ^19^ y los patrones de crecimiento de la OMS. Según el IMC, se consideró: delgadez, como uno menor de 2 desviaciones estándar (DE); riesgo de delgadez, entre -2 y <1 DE, adecuado para la edad, entre -1 y 1, sobrepeso, entre >1 y 2, y obesidad, como >2 DE.

En cuanto a los patrones de actividad física de los escolares con sobrepeso y obesidad, se determinó la frecuencia como diaria, semanal o mensual. Se estableció el número de veces por semana y el tiempo frente a las pantallas de televisión, computador o videojuegos. En general, la actividad física se categorizó como: “casi nunca”, si era menos de dos veces por semana; “algunas veces”, si era más de dos veces por semana, y “siempre”, si era salir a algún tipo de actividad, por lo menos, una hora tres veces a la semana.

La captura de los datos se hizo con el programa Epi Info 7. En el análisis de los datos descriptivos e inferenciales, se usó el programa IBM SPSS™, versión 23. Se hizo el análisis bivariado para las variables continuas buscando diferencias significativas mediante la prueba t, con un nivel de significación de p<0,05. Se utilizó la prueba de ji al cuadrado para comparar las proporciones y para aceptar la hipótesis nula, considerando un nivel de significación del 5 % para una mayor confianza en los resultados.

### Consideraciones éticas

El estudio contó con la aprobación del Comité de Ética de la Universidad Libre, seccional Cali, además de la autorización del rector y los directivos y la colaboración de los profesores de la institución educativa, así como de los padres que aceptaron la participación de sus hijos, a quienes se les leyó y explicó el documento del consentimiento informado para su posterior su firma.

Se cumplió el principio bioético de protección, respeto y autonomía de los niños participantes con la firma del documento de asentimiento informado una vez lo comprendieron y se resolvieron todas sus inquietudes. Se respetó, asimismo, la privacidad de los datos.

## Resultados

La población estudiada incluyó 150 menores, 96 niños y 54 niñas, de una institución educativa privada; el 40 % estaba en primer grado y la edad promedio era de 8 años. El 78 % pertenecía a los niveles socioeconómicos 4 y 5. El rango de edad más frecuente en los padres fue entre los 40 y los 50 años para ambos sexos: padres, 55 %, y madres, 57 %. En cuanto al número de hermanos, el 47 % tenía un solo hermano y el 25 % correspondía a hijos únicos. En lo que respecta al nivel de escolaridad de los padres, el 76 % de las madres y el 70 % de los padres eran profesionales; el 80 % de las madres y el 90 % de los padres trabajaban fuera del hogar (cuadro 1).

**Cuadro 1:** Caracterización sociodemográfica de la población total de estudio (N=150)

Características de los niños y padres encuestados		n	(%)
Sexo			
	Masculino	96	64
	Femenino	54	36
Estrato		31	21
	3	47	31
	4	70	47
	5	2	1
	6		
Grado escolar del niño			
	Primero	45	30
	Segundo	21	14
	Tercero	33	22
	Cuarto	26	17
	Quinto	25	17
Edad del padre (años)			
	35 a 40	36	24
	41 a 50	83	55
	51 a 59	14	9
	>60	17	11
Edad de la madre (años)			
	35 a 40	32	21
	41 a 50	85	57
	51 a 59	18	12
	>60	15	10
Número de hermanos			
	Ninguno	38	25
	Al menos uno	71	47
	2 o más	41	27
Ocupación de la madre			
	Trabajo en casa	30	20
	Trabajo fuera de casa	120	80
Escolaridad de la madre			
	Profesional	114	76
	No profesional	36	24
Ocupación del padre			
	Trabajo en casa	15	10
	Trabajo fuera de casa	135	90
Escolaridad del padre			
	Profesional	105	70
	No profesional	45	30
Diagnóstico nutricional			
	Delgadez	1	1
	Normal	75	50
	Obesidad	20	13
	Riesgo de delgadez	12	8
	Sobrepeso	42	28
Actividad física			
	Casi nunca	7	5
	Algunas veces	108	72
	Siempre	35	23

En cuanto al diagnóstico nutricional, se encontraron 42 niños con sobrepeso, 20 con obesidad y el 50 % de la población tenía un estado nutricional normal. La prevalencia de sobrepeso y obesidad de toda la población fue del 41 %, la frecuencia total de sobrepeso era del 28 % y la de obesidad del 13 %; el 8 % de los niños estaba en riesgo de delgadez y el 1 % presentaba delgadez.

La frecuencia del consumo de alimentos se determinó como diaria, semanal y mensual, y los datos obtenidos se compararon con los de la ENSIN 2015 (6) por periodicidad diaria. Entre los alimentos de mayor consumo diario, se encontraron cereales y sus derivados (91 %), jugos de caja y gaseosas (88 %), y dulces y golosinas (88 %). Los grupos de alimentos que no se consumían de manera diaria incluyeron vegetales (93 %), hígado y vísceras (93 %), aceite y grasas (84 %), carne, pollo y pescado (59 %) (cuadro 2).

**Cuadro 2:** Frecuencia diaria de consumo de los alimentos en los escolares comparados con la ENSIN 2015

Alimentos	Consumo diario escolares (%)	Consumo diario ENSIN/2015 (%)
Leche y derivados lácteos	66	91,0
Carnes (pollo, pescados)	41	94,0
Huevos	61	97,0
Vísceras	7	33,0
Frutas	16	85,0
Verduras	7	69,9
Dulces y golosinas	88	89,0
Paquetes	47	82,5
Embutido	32	78,0
Comida chatarra	45	57,5

En el cuadro 2 aparecen los resultados que se compararon con los de la ENSIN 2015 ^6^. Se encontró un consumo muy bajo de vísceras (7 %) comparado con el porcentaje para Colombia (33 %); un consumo de frutas del 16 % comparado con el 85 % en la encuesta nacional y un consumo diario de verduras del 7 % frente al 69,9 % nacional, lo que refleja los inadecuados hábitos alimentarios y el consumo de pocos alimentos ricos en micronutrientes. Se encontró consumo de comida chatarra en el 45 %, de paquetes en el 47 %, y de embutidos en el 32 %, comparado con el 57,5, el 82,5 y el 78 %, respectivamente, del consumo diario registrado en la ENSIN 2015 ^6^. La prevalencia de sobrepeso y obesidad presentaron una alta correlación con el consumo diario de cereales y derivados (91 %), y jugos de caja y gaseosas (88 %); además, se encontró una fuerte asociación entre el sobrepeso y el consumo de jugos naturales (68 %). También, se pudo observar un consumo diario de lácteos del 55 %, siendo más bajo que el de la ENSIN 2015 (91 %) ^6^ (cuadro 2).

En el análisis de correlación, que mide la asociación de interdependencia entre las variables relacionadas (cuadro 3), se encontró una relación con el número de hermanos, es decir, los escolares con mayor prevalencia de sobrepeso u obesidad tendían a ser hijos únicos o a tener solo un hermano; también, se asociaron la edad de la madre, en especial cuando era mayor de 50 años, la escolaridad de los padres, su ocupación o trabajo fuera del hogar, y la pertenencia a los estratos socioeconómicos 4 y 5.

**Cuadro 3:** Asociación entre las variables sociodemográficas, y el sobrepeso y obesidad

Variable		Niños con sobrepeso u obesidad	n	(%)
Sexo				
	Niños	39	63	0,842
	Niñas	23	37	
Grado				
	Primero	25	40	0,234
	Segundo	9	15	
	Tercero	13	21	
	Cuarto	7	11	
	Quinto	8	13	
Edad de la madre				
	35 a 41	12	19	0,035
	41 a 50	40	65	
	51 a 59	5	8	
	> 60	5	8	
Número de hermanos				
	Ninguno	21	34	0,001
	Al menos uno	22	35	
	2 o más	19	31	
Ocupación del padre				
	Trabajo en casa	13	21	
	Trabaja fuera de casa	49	79	0
Escolaridad del padre				
	No profesional	18	29	
	Profesional	44	71	
Ocupación de la madre			31	0,756
	Trabajo en casa	19	69	
	Trabajo fuera casa	43		
Escolaridad				
	No profesional	20	33	
	Profesional	42	67	
Estrato				0,025
	3	16	26	
	4	23	31	
	5	21	47	
	6	2	3	

La relación con la edad de la madre (p=0,035) fue significativa (cuadro 4), así como con el número de hermanos (p=0,001), que fue significativa cuando no había hermanos o solo uno. También, hubo asociación con los alimentos de mayor consumo diario y semanal, y en cuanto al consumo diario de lácteos, fue tan solo del 66 %, siendo muy bajo en comparación con lo registrado en la ENSIN 2015 (91 %) ^6^.

**Cuadro 4:** Correlación y evidencia entre la asociación del sobrepeso y la obesidad con las variables estudiadas

Variable	p	Conclusión
Edad de la madre	0,035	Existe evidencia para aceptar que hay asociación entre el sobrepeso y la edad de la madre.
Sexo del estudiante	0,842	No hay evidencia para aceptar que exista asociación entre el sobrepeso y el sexo del menor.
Número de hermanos	0,001	Existe evidencia.
Consumo de cereales	0,0246	Existe evidencia.
Comidas rápidas	0,0367	Existe evidencia.
Jugos empacados y jugos naturales	0,001	Existe evidencia.
Grasas	0,0191	Existe evidencia.
Azúcares	0,000	Existe evidencia.
Profesión de la madre	0,756	No existe evidencia.
Profesión del padre	0,001	Existe evidencia.

En lo referente a los patrones de actividad física, definida como salir a ejercitarse de alguna manera, por lo menos, una hora tres veces a la semana, se encontró que el 95 % de los niños casi nunca lo hacían (cuadro 5). La actividad física más frecuente fue montar en bicicleta (80 %), pero menos de dos veces por semana; nadar (77 %), aunque el 68 % lo hacía menos de dos veces por semana, y saltar o correr (73 %), pero 45 % de ellos lo hacía menos de dos horas y solo el 21 % más de cinco veces por semana. En cuanto a los deportes con importante gasto calórico, como el baloncesto y el fútbol, los niños los practicaban menos de dos veces por semana: baloncesto (53 %) y fútbol (37 %).

**Cuadro 5:** Actividad física y dedicación (número de veces) por semana en los escolares

Actividad	Nunca 1 a 2				3 a 4 >5			
	n	%	n	%	n	%	n	%
Saltar la cuerda	25	41	32	51	1	1	4	7
Patinaje en línea	41	66	19	31	2	3		0
Caminar como ejercicio	42	67	19	31		0	1	2
Montar bicicleta	12	20	45	72	1	1	4	7
Saltar o correr	17	27	28	45	4	6	13	21
Ejercicios aeróbicos	41	66	19	31	1	1	1	2
Nadar	14	23	42	68	6	9		0
Bailar	32	51	30	49		0		0
Patinar en monopatín	37	59	23	37	1	1	1	2
Jugar fútbol	23	37	23	37	6	9	10	6
Jugar voleibol	23	37	23	37	6	9	10	17
Jugar baloncesto	28	45	33	53	1	2		0
Artes marciales (karate, taekwondo)	42	67	18	29	1	1	1	2

**Figura 1:**
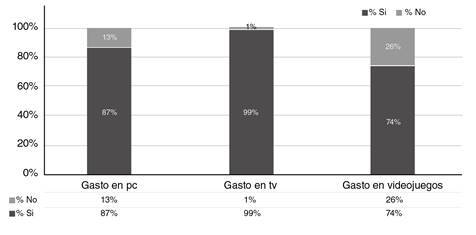
Frecuencia en gasto de tiempo frente a pantallas de computador, televisión y videojuegos

**Figura 2:**
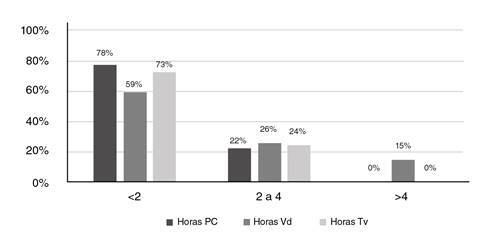
Frecuencia y número de horas frente a pantallas de computador, televisión y videojuegos

También, se encontró que el 87 % dedicaba tiempo al computador, el 99 % veía televisión y el 74 % practicaba videojuegos (figura 1). Con respecto al número de horas dedicadas a las pantallas, el 78 % permanecía en promedio menos de dos horas frente a estas, cifras que supera la de la última encuesta para Colombia (6), con una prevalencia del 67 %. El 26 % y el 24 % de los niños dedicaba más tiempo a los videojuegos y la televisión: entre 2 y 4 horas, respectivamente, es decir, el porcentaje de niños que pasaba tiempo frente a las pantallas fue alto, pero al preguntar por el número de horas al día, estas cifras disminuían (figura 2).

## Discusión

La prevalencia encontrada del 41 % de sobrepeso y obesidad se considera demasiado alta, y obliga a plantear estrategias de intervención con base en los datos reales, actualizados y concretos de la región para modificar los estilos de vida de la población infantil.

En cuanto a las características familiares, los niños con exceso de peso pertenecían a los estratos socioeconómicos 4 y 5 (78 %), lo que demuestra, como lo han mencionado otros estudios ^20^, que los mayores ingresos favorecen o se asocian con sobrepeso y obesidad, así como la asociación con el número de integrantes de la familia y con ser hijo único o tener solo un hermano ^1^.

Este hallazgo es importante porque en diversos estudios se han registrado datos controvertidos en cuanto al hecho de que el “tener familias con menos de cinco integrantes representa un mayor riesgo de sufrir exceso de peso” ^21^^-^^23^, pues los padres con mayores posibilidades de adquisición y distribución de recursos para el hogar y las madres con edades entre los 41 y los 50 años, tienen mayor posibilidad de que sus hijos tengan exceso de peso, como lo evidenciaron los resultados de este estudio.

Asimismo, influyeron el nivel socioeconómico, el número de integrantes del hogar, la escolaridad y la ocupación de los padres: el 71 % de los padres eran profesionales y el 79 % de las madres trabajaba fuera de casa, condiciones que limitan el tiempo de que disponen para el cuidado de los hijos, pues deben dejarlos a cargo de otro adulto, como lo estableció un estudio sobre los estilos de crianza y su relación con la obesidad ^1^. Este aspecto podría impedir que las madres promuevan en su hijo las preferencias y puntos de vista que favorecen su crianza, aunque en el presente estudio se consideró que esta hipótesis debería comprobarse. Esto, aunado al sedentarismo de gran parte de los menores estudiados, que no practicaban actividades físicas con gasto calórico importante, o lo hacían muy poco, favorece la aparición del exceso de peso.

En resumen, el presente estudio encontró una prevalencia de obesidad en la población estudiada del 13 % frente al 7,6 % de la ENSIN 2015 ^6^. La proporción de sobrepeso fue del 28 % frente al 16,8 % en la ENSIN 2015 ^6^, lo que indica una elevación del 67 % en este mismo grupo de edad. El total del exceso de peso fue del 41 % frente al 24,4 % en la ENSIN 2015 ^6^, es decir, un incremento del 170,8 % que, a pesar del tamaño reducido de la muestra, ubica al país como una de las regiones con cifras más elevadas de exceso de peso en este grupo de edad en América, y alerta sobre las comorbilidades que pueden presentarse a mediano y largo plazo como consecuencia de esta alta prevalencia ^22^^,^^23^. Las variaciones en cuanto a las prevalencias se registran en diversas regiones del mundo, por ejemplo, en México, la población en edad escolar ha presentado valores de sobrepeso y obesidad del 34,4 % ^24^.

En cuanto al índice de riqueza en estratos medios y altos, según la ENSIN 2015 ^6^, la prevalencia fue del 31,6 %, muy por debajo de las cifras encontradas en este estudio, lo que significa que estos porcentajes en escolares de Cali siguen siendo muy elevados y muy por encima de otros países latinoamericanos ^22^^,^^25^, lo que obliga a revisar las estrategias de intervención en esta etapa del ciclo vital. En uno de los últimos estudios realizados en el departamento del Valle, ^26^, en una muestra de 1.789 escolares de instituciones del sector público, se encontró una prevalencia del 14,7 % de sobrepeso y del 6,3 % de obesidad, valores por debajo de la mitad de los datos encontrados en este estudio. Tarqui-Mamani, *et al*. ^22^, encontraron que un mayor nivel socioeconómico o la ausencia de la condición de pobreza se asociaban con una mayor prevalencia de sobrepeso y obesidad.

Por otra parte, la estructura familiar de un solo hijo o pocos miembros en la familia se asoció con la prevalencia del exceso de peso, lo que coincide con los resultados de un estudio en Perú ^22^, pero difiere del todo de lo hallado en uno realizado en Cartagena, Colombia ^21^, en el que se evidenció que un mayor número de integrantes en la familia disminuía el riesgo de exceso de peso. De todas maneras, se encontró que los abuelos son quienes más tiempo dedican al cuidado de los niños y, en otros casos, estos viven con, al menos, uno de los abuelos, lo que puede contribuir a comportamientos que propician la obesidad e implicaría una influencia negativa del entorno familiar, ya que los comportamientos de salud de los niños pueden ser modelados por los patrones de los padres y los demás miembros de la familia ^27^^,^^28^.

En otro estudio en Argentina ^29^ entre escolares pertenecientes a un estrato socioeconómico alto, se encontró una mayor prevalencia de sobrepeso y obesidad a medida que el índice de riqueza aumentaba, y lo mismo ocurrió en varios estudios en Brasil ^30^^,^^31^ en los que se concluyó que, a pesar de que el sobrepeso y la obesidad aumentaron en todas las clases sociales, fue mayor en los hogares con más poder económico. Según otros estudios ^1^^,^^32^, las condiciones económicas de las familias aparecen como un factor asociado con la obesidad infantil, así como las formas de autoridad de los padres, que, de no modificarse, propiciarían conductas en el niño que persistirían, incluso, en la adultez.

Es importante revisar y ampliar los estudios sobre el sobrepeso y la obesidad en el contexto familiar, pues allí se desarrollan los hábitos alimentarios. Deben analizarse los factores paternos y socioculturales, la relación con la ocupación de los padres y su escolaridad, y la forma como les explican a sus hijos sus eventuales problemas de peso e inciden en su dieta, para así comprender el efecto en el riesgo de obesidad. Como se menciona en el estudio de Montiel, *et al*. ^1^, deben evaluarse otras características de las familias, entre ellas, el apoyo social al niño, la posibilidad de un trabajo estable de los padres y, especialmente en el caso de la madre, uno que le permita estar en casa. También, debería indagarse sobre las costumbres de los padres y las circunstancias que inciden en su adquisición, así como las estrategias que utilizan las madres para manejar las prácticas de alimentación ^33^.

Al analizar la frecuencia de consumo en el grupo de escolares estudiados, se encontró una proporción de ingestión diaria de huevo (61 %) menor de la reportada en la ENSIN 2015 (97 %) ^6^. Según las guías alimentarias de Colombia ^34^, se recomienda incluir el huevo en la dieta diaria, ya que en los metaanálisis se han demostrado sus beneficios. Se le considera un constituyente esencial en la dieta de las personas en todas las etapas de la vida y podría tener un papel aún más importante en aquellas con mayores necesidades nutricionales, como la infancia, el embarazo y la tercera edad ^35^^,^^36^. Se encontró, asimismo, un consumo de lácteos y derivados del 66 %, por debajo del promedio nacional del 91 %. La ausencia de algunos alimentos esenciales en el menú diario tendría como consecuencia el déficit de algunos micronutrientes.

En cuanto al consumo de vísceras, se encontró una proporción tan solo del 7 %, muy por debajo del promedio registrado en la ENSIN 2015(33 %) ^6^. Esto es muy relevante, si se tiene en cuenta la importancia de estos alimentos por su alto contenido de hierro, sobre todo el de origen animal, y su aporte en la disminución de la prevalencia de anemia, la cual es del 8 % en estos grupos de edad en el país ^6^. En el estudio, el consumo de carnes rojas se daba en el 79 % de los niños, muy por debajo del promedio nacional (93,5 %) y de lo hallado en otros estudios ^37^. Debe anotarse que, aunque hay controversia sobre el consumo diario de carnes rojas, en salud pública se recomienda disminuir su consumo, pero no eliminarlas de la dieta dado su gran valor nutricional como fuente de aminoácidos esenciales difíciles de cubrir solo con alimentos de origen vegetal.

El promedio de consumo de pescado en nuestro estudio fue del 71,5 %, por encima del 54,5 % reportado en la ENSIN 2015 ^6^. Se sabe que el pescado contiene nutrientes como el cinc y, en algunos casos, el omega 3, esenciales para el sistema cardiovascular y, dado que el organismo no los sintetiza, es importante su consumo en la dieta diaria ^38^.

También, se encontró que el 91 % de los niños del estudio no consumían verduras y la proporción del consumo de frutas era del 68 %, muy por debajo del promedio nacional del 69,9 y 85 %, respectivamente, en tanto que la proporción de la ingestión de dulces y golosinas estuvo muy cerca de dicho promedio (88 %). Este es un elemento que contribuye al aumento del contenido calórico de la dieta diaria y tiene una asociación positiva con la aparición del sobrepeso y la obesidad ^39^. Aunque los carbohidratos constituyen, por lo menos, las dos terceras partes de la alimentación, se recomienda controlar su consumo, especialmente de los simples, y aumentar el de los carbohidratos complejos ^40^. Por otra parte, el consumo de comidas rápidas fue del 45 %, por debajo del promedio nacional del 57,5 %, lo que permite concluir que, aunque se trataba de un grupo con recursos suficientes, estas no hacían parte de la dieta diaria de un porcentaje importante de los niños.

El consumo de comidas de paquete estuvo muy por debajo del promedio nacional, con una proporción del 47 % frente al 82,5 %, es decir que a pesar de las prevalencias elevadas de sobrepeso y obesidad en este grupo y su comprobada asociación con el consumo de alimentos empaquetados ^41^, en el estudio no se observó dicha relación; además, en el colegio no se ofrecían estos alimentos a los estudiantes.

En la etapa escolar, un período en el que se establecen de manera definitiva los hábitos alimenticios y se consolidan las preferencias de alimentos en la dieta y las pautas del posterior comportamiento nutricional en la vida adulta, es importante hacer el seguimiento e intervenir adecuada y oportunamente para evitar posibles desviaciones y promover buenos patrones de consumo para la adultez. Se sabe que uno de los riesgos de la obesidad infantil radica en el desarrollo de comorbilidades y que el exceso de peso en los primeros cinco años de vida puede mantenerse hasta llegar a la edad adulta en 20 % de los niños, en tanto que, en los adolescentes, este se mantiene hasta en el 80 %, con la consecuente elevación de las tasas de morbilidad y mortalidad por enfermedades cardiovasculares y otras ^42^^,^^43^^).^

Los profesores también tienen un papel importante en el establecimiento de los hábitos alimenticios, ya que el niño pasa gran parte del día en el colegio. Por ello, los centros educativos deben ser promotores de salud con base en las estrategias internacionales sobre alimentación saludable ^44^. En las tiendas escolares deben promoverse los cambios en los hábitos y ofrecer alimentos de calidad nutricional; además, debe incentivarse la práctica de actividades físicas adecuadas en las escuelas.

Los resultados del estudio pueden parecer controversiales frente a los de otros en que el sobrepeso y la obesidad eran mayores en los estratos socioeconómicos bajos por su mayor consumo de carbohidratos y grasas, menor consumo de frutas y verduras, y poca actividad física ^45^. Una posible explicación sería el mayor poder adquisitivo, que permite más acceso y consumo de alimentos y más dinero para la compra de videojuegos y pantallas, lo que contribuye al sedentarismo. En cuanto a la escolaridad de padre y madre conjuntamente, no hubo asociación significativa, pero sí la hubo (p<0,001) con la escolaridad paterna y con el trabajo fuera de casa (79 % de los padres y 69 % de las madres), lo que coincide con otros estudios publicados ^1^. La ocupación informal del jefe del hogar se relacionó con una menor prevalencia de sobrepeso, dato controversial como el de la asociación de la educación o nivel de escolaridad de los padres con el estado nutricional de los hijos ^46^.

A pesar del tamaño reducido de la muestra en el presente estudio, al comparar los resultados con los de la ENSIN 2015 ^6^, se encontró que en el mismo grupo de edad la proporción del exceso de peso ha variado hacia el ascenso, lo que indica que debería dársele prioridad. En el 2010, la preocupación más importante en cuanto a las alteraciones relacionadas con la alimentación en la infancia era la desnutrición, pero los actuales cambios en el perfil epidemiológico de las poblaciones, el mayor nivel de ingreso y la disminución de la actividad física deben tenerse en cuenta a la hora de evaluar el sobrepeso y la obesidad ^22^.

En este sentido, es muy importante realizar más estudios representativos en diferentes regiones del país para determinar la prevalencia de sobrepeso y obesidad, y ahondar en las causas que la determinan, con el fin de diseñar intervenciones directas y establecer medidas de prevención primaria orientadas a las causas específicas del problema.

Con respecto a la actividad física, se encontró que el 67 % de los niños ni siquiera cumplía con los niveles mínimos (caminar como ejercicio tres veces a la semana durante una hora o 180 minutos a la semana), es decir, el sedentarismo estaba afianzado y pasaban mucho tiempo frente a las pantallas, sin actividad física que implicara un gasto calórico importante, lo que, comparado con la ENSIN 2015(6), estuvo muy lejos del promedio nacional. Algunos de los participantes en el estudio hacían ejercicio como saltar, correr o jugar baloncesto, pero sin la periodicidad en horas recomendada (cuadro 5).

El tiempo empleado frente a las pantallas fue muy parecido al que prevalecía en el país ^6^, con cifras elevadas de inactividad física y sedentarismo ^47^, loque aumenta el sobrepeso y la obesidad, por lo que debe intervenirse ^48^. Es imprescindible una suficiente disponibilidad de espacios y oportunidades para la actividad física regular en el entorno escolar, pues los niños en estas etapas pasan gran parte del día en la escuela ^49^. Sin embargo, la práctica de educación física en las escuelas primarias ha disminuido a pesar de las evidencias sobre sus beneficios y las recomendaciones de la OMS ^49^^,^^50^. Por ello, es necesario que las entidades gubernamentales y no gubernamentales intervengan para extender el número de días y horas a la semana dedicadas a la educación física en las escuelas y jardines infantiles ^51^.

Estos resultados corroboran que el sobrepeso y la obesidad están alcanzando niveles muy elevados en los escolares colombianos y que existe la urgente necesidad de trabajar en grupos interdisciplinarios en torno a cada uno de los factores asociados con el problema, como la alimentación desequilibrada, la actividad física y algunos factores familiares ^12^. Con los datos específicos que se presentan, puede aproximarse un diagnóstico de la situación actual de la prevalencia del sobrepeso y la obesidad infantiles en este nivel socioeconómico en Cali, y proponer nuevos estudios en la ciudad y el departamento.

## Conclusiones

Se concluyó que la prevalencia de sobrepeso y obesidad en la población de estudio fue del 41 %: 28 % de sobrepeso y 13 % de obesidad. Se encontró asociación entre algunas variables, como: el número de hermanos (los escolares con mayor exceso de peso tendían a ser hijos únicos o tener solo un hermano); la edad de la madre, especialmente entre los 41 y los 50 años; la escolaridad de los padres y la ocupación o trabajo fuera del hogar, así como la pertenencia a los estratos socioeconómicos 4 y 5. El consumo de lácteos y derivados y el de vísceras estuvieron por debajo del promedio nacional (7 %). El 91 % de los niños consumía verduras, pero solo el 68 % comía frutas en la dieta diaria. Se evidenció un alto consumo de dulces y golosinas. El 67 % no hacía actividad física mínima y pasaba mucho tiempo frente a las pantallas, y el ejercicio con gasto calórico importante era muy limitado.

Se recomiendan los programas de prevención y promoción contra el sobrepeso y la obesidad infantil, sobre todo en los niños en etapa escolar, con diferentes estrategias que propicien una alimentación balanceada y de calidad nutricional, y más tiempo y mayor variedad de actividades físicas y estímulos para conservar el peso adecuado para la edad y la talla, sin llegar a excesos de preocupación por la imagen corporal. En el ámbito escolar, deberían aumentarse las sesiones de actividad física y el desarrollo de habilidades fundamentales de movimiento. Debe procurarse un suministro de alimentos balanceados con una distribución adecuada de macronutrientes y micronutrientes, así como prácticas culturales que incentiven la alimentación saludable y mejores estilos de vida de los niños. Además, debe asegurarse el acceso a gimnasios o campos deportivos en los planteles educativos.

También, debe trabajarse con padres, abuelos, otros familiares y cuidadores, en el mejoramiento de su nivel de actividad física para que sean un ejemplo, y organizar talleres de alimentación saludable y actividades de apoyo en casa que animen a los niños a estar más activos y a pasar menos tiempo frente a las pantallas.

En futuros estudios debe enfatizarse en la descripción de los estilos parentales y de cuidadores, y hacer prácticas de control en el campo para establecer conductas saludables que eviten el desarrollo de la obesidad; además, trabajar en la elaboración de guías de manejo fáciles de aplicar a cargo de profesionales en el manejo integral del niño.
